# 3t-seq: automatic gene expression analysis of single-copy genes, transposable elements, and tRNAs from RNA-seq data

**DOI:** 10.1093/bib/bbae467

**Published:** 2024-09-25

**Authors:** Francesco Tabaro, Matthieu Boulard

**Affiliations:** Epigenetics and Neurobiology Unit, EMBL Rome, European Molecular Biology Laboratory, Via Ercole Ramarini 32, Monterotondo 00015, Italy; Epigenetics and Neurobiology Unit, EMBL Rome, European Molecular Biology Laboratory, Via Ercole Ramarini 32, Monterotondo 00015, Italy

**Keywords:** RNA-seq data analysis, differential gene expression analysis, single-copy genes, transposable elements, tRNA, Snakemake pipeline

## Abstract

RNA sequencing is the gold-standard method to quantify transcriptomic changes between two conditions. The overwhelming majority of data analysis methods available are focused on polyadenylated RNA transcribed from single-copy genes and overlook transcripts from repeated sequences such as transposable elements (TEs). These self-autonomous genetic elements are increasingly studied, and specialized tools designed to handle multimapping sequencing reads are available. Transfer RNAs are transcribed by RNA polymerase III and are essential for protein translation. There is a need for integrated software that is able to analyze multiple types of RNA. Here, we present 3t-seq, a Snakemake pipeline for integrated differential expression analysis of transcripts from single-copy genes, TEs, and tRNA. 3t-seq produces an accessible report and easy-to-use results for downstream analysis starting from raw sequencing data and performing quality control, genome mapping, gene expression quantification, and statistical testing. It implements three methods to quantify TEs expression and one for tRNA genes. It provides an easy-to-configure method to manage software dependencies that lets the user focus on results. 3t-seq is released under MIT license and is available at https://github.com/boulardlab/3t-seq

## Introduction

RNA-seq has become the standard method to quantify differential gene expression in biological systems. Robust, reproducible data analysis workflows are critical to ensure comparable results across experiments [1, 2]. The vast majority of available pipelines are tailored for the analysis of the expression of single-copy genes. For example, the ENCODE consortium released their pipeline [3] as an open-source package on GitHub; similarly, the NASA GeneLab pipeline [4] implements a workflow based on TrimGalore [5], STAR [6], RSEM [7], and DESeq [8]. The VIPER pipeline [9] is a fully automatic implementation of best practice guidelines. The nf-core community [10] produced a pipeline implementing multiple combinations of tools for single-copy genes analysis. Additional pipelines couple gene expression quantification with other types of questions. For example, SPEAQeasy [11] bundles standard steps with variant calling; RNAflow [12] joins gene expression quantification with *de novo* transcriptome assembly using Trinity [13], BUSCO [14], and StringTie [15]. Other tools were created to address alternative splicing [16–18], gene fusion events [19–23], circular RNA expression [24], or analysis of time series data [25]. Some of the tools mentioned are explicitly targeted to novice users, providing graphical user interfaces [16, 17].

While the analysis of RNAs transcribed by single-copy genes is well established, the analysis of transposable elements (TEs) expression is more challenging because of their repetitive nature, which makes the genomic assignment of short reads difficult. In addition, their analysis requires separate genome annotations, such as Repeat Masker [26]. TEs and tandem repeats (e.g. satellite DNA) are epigenetically silenced in physiological conditions but can be transcribed in specific situations, resulting in sequencing reads that map to multiple genomic locations. This feature, coupled with the reduced length of sequencing reads from common methods, results in ambiguous genome mapping. Traditional pipelines for RNA-seq analysis discard ambiguous reads as they inflate gene expression estimation. Specialized tools have been created to account for such reads and quantify expression of TEs and other repeated sequences. For example, TEtranscripts [27] solves this problem by assigning reads to TEs families using expectation–maximization. However, TEtranscripts require genomic alignments as input, which need to be handled with external tools. On the other hand, TEtools [28] implements genome mapping, but it does not quantify single-copy gene expression focusing explicitly on TEs. In recent years, two automatic pipelines were created to integrate the analysis of single-copy genes and TE. For example, GeneTEFlow [29] uses the SQuiRE software [30] to estimate locus-specific expression of TEs. Finally, in a benchmark experiment [31], a workflow combining STAR [6] and FeaturesCount [32] has been recently shown to yield more accurate expression estimates than Bowtie2 [33] and TEtranscripts [27].

None of the tools described so far include gene expression analysis of tRNAs. This class of genes is of fundamental importance for all organisms because their transcripts are required for protein synthesis. While standard genomic annotations report tRNA features, specialized predictive models have been developed to improve on them with more accurate localizations, better functional classification, and more accurate discrimination between actual tRNA genes and tRNA-derived repetitive elements [34]. These predictions are collected in gtRNAdb [35].

Here, we present 3t-seq, an implementation of best practices for integrated differential expression analysis of single-copy genes, TEs, and tRNAs from RNA-seq data. We leverage specialized annotations for each molecular species and implement an automated Snakemake [36] workflow, allowing standardized analysis of multiple sequencing libraries while aiming at increasing reproducibility, scalability, and portability. By bundling analysis of multiple RNA species, we ensure that all analyses are executed in homogeneous software environments. We remove the possibility of using different versions of the same software, and we enforce utilization of the same genomic reference files for the entire analysis cycle. In addition, our pipeline delivers a report collecting running statistics, quality control metrics, and interactive visualizations to simplify results interpretation and hypothesis generation.

## Materials and methods

### Implementation details

**Figure 1:**
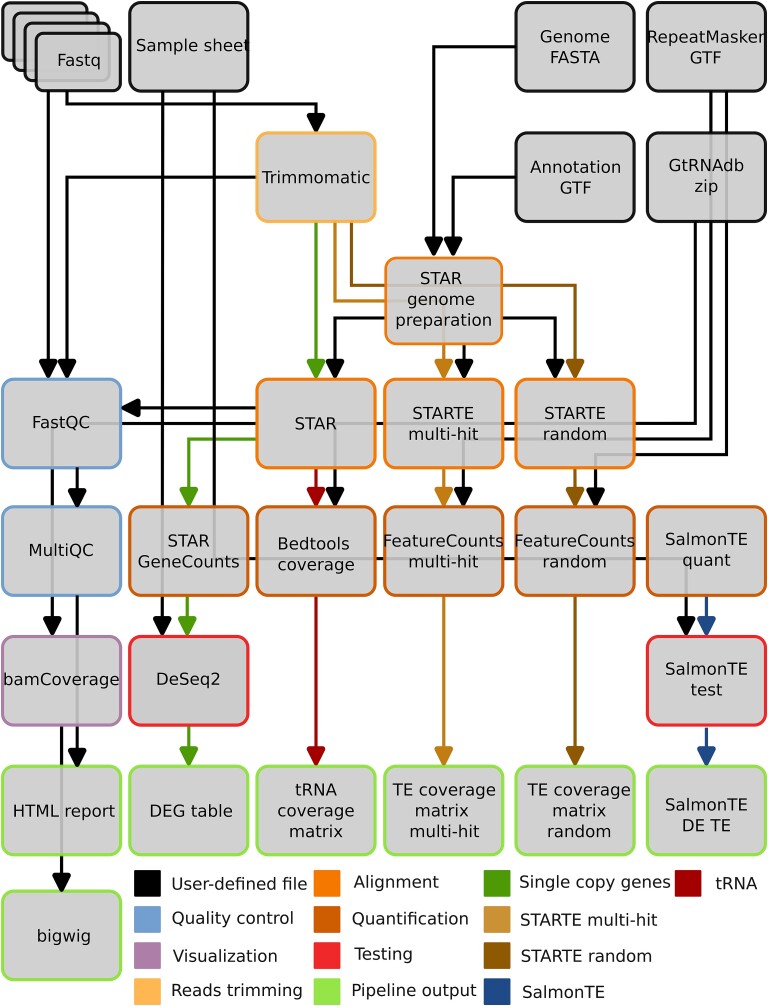
Schematic representation of 3t-seq workflow. Pipeline inputs are fastq files, a sample sheet and genome annotation files. Input fastq files are trimmed and fed into multiple instances of the STAR aligner to generate alignment files and gene expression quantification files. Gene expression quantification files are then used to calculate differential gene expression and generate lists of differentially expressed genes. Final outputs include FastQC, MultiQC HTML reports, tables of differentially expressed genes, read counts matrices (for protein coding genes, TE and tRNA), SalmonTE tables and figures.

The pipeline is implemented as a Snakemake [36] workflow (Fig. 1). This framework ensures the correct execution order, resources allocation, and parallelization of analysis steps while enabling dependencies management and correct environment initialization. It supports execution in different computing environments ranging from standard laptop and desktop machines to server, high-performance, and cloud computing environments. The pipeline was developed and tested on machines running standard enterprise-grade Linux distributions. In addition, the Snakemake framework allows for integrated dependencies management and generation of a self-contained, interactive HTML report after each run. The 3t-seq pipeline builds on this feature and implements a series of interactive tables and plots to readily visualize results of the final steps (differential expression analyses).

3t-seq inputs are raw sequencing data in Fastq format and a comma-separated table describing sample attributes. The pipeline requires a reference genome sequence, its associated annotation file, and a tRNA annotation file. Given remote URLs to these files, the pipeline can download and store them locally automatically. The pipeline uses the University of Californa Santa Cruz (UCSC) Genome Browser RESTful Application Programming Interface (API) to download a JavaScript Object Notation (JSON) representation of the RepeatMasker track and convert it into Gene Transfer Format (GTF). In case that custom genome builds are stored on the local file system, the pipeline can be configured to use these files instead.

Both total and polyA-enriched libraries sequenced with single-end and paired-end protocols are supported. For analysis of three types of molecular species, the input is ribo-depleted total RNA-seq data. 3t-seq also supports all variations of mRNA-seq (stranded, Smart-seq…) and does not discriminate against the RNA isolation protocol.

### Quality control and preprocessing

The initial quality control checks are carried out with FastQC [37] and the results collected in a report using MultiQC [38]. Reads are trimmed with Trimmomatic [39]. The pipeline allows users to specify custom trimming strategy via the pipeline config file. Users need to specify the trimming strategy according to the Trimmomatic manual. Trimming results are fed into FastQC, and an overview report is generated with MultiQC to allow comparison of results before and after the procedure.

All mappings to reference genome are performed with STAR [6]. Similarly to the trimming step, users can define custom options, if needed. For single-copy genes, the mapping procedure generates alignments both in genomic coordinates and transcriptomic coordinates. Picard’s MarkDuplicates [40] is used to mark duplicate reads. Resulting files are readily converted to bigwig enabling faster genome browser–based visualization. Alignment and duplicate marking statistics are aggregated in an interactive report with MultiQC.

For TEs, trimmed reads are mapped with STAR but with different sets of parameters. Recently, a comparative study [31] identified two sets of parameters as best correlating with a known expression level in synthetic and natural datasets. These parameters are included in 3t-seq. Briefly, one set allows reads to map to a high number of loci and all of them are kept in the output alignment. The other randomly selects only one mapping location for each multimapper. Different alignment strategies have implications in TEs quantification downstream, as discussed below. We termed these two methods STARTE-multi and STARTE-random.

### Gene expression quantification and differential expression testing

To quantify gene expression, STAR’s geneCounts quantification mode is enabled. Gene counts are collected in a matrix. To assess differential expression, DESeq2 [8] is used. Users can configure the pipeline to test for differential expression of a specific experimental variable that has to be provided in the sample sheet.

TEs expression is quantified with FeatureCounts [32]. In STARTE-multi configuration, factional quantification is enabled. This accounts for multimapping reads by weighting their score by the number of loci it maps to; in other words, any multimapping read contributes to the expression of a given TE by $1/\mathrm{x}$ where $\mathrm{x}$ is the number of loci it maps to. On the other hand, in STARTE-random, multimapping reads are randomly assigned to one of its target loci; therefore, a unary scoring function can be used and its contribution to the TE expression can be counted as one.

The pipeline also runs SalmonTE [41] to quantify and test for differential expression of TEs in an alignment-free manner. This tool uses Salmon [42] and DESeq2 [8]. The process is split into two parts: the first quantifies TEs expression producing a counts table summarized by TE clade. The second tests for differential expression between samples and outputs a table with ${\log}_2$ fold change, ‘*p-*value’ and statistics value together with visualizations.

In addition, 3t-seq assesses the expression of tRNAs by looking at the coverage of tRNA genes from gtRNAdb [35]. Coverage values are computed using Bedtools [43]. Starting from the alignments computed for single-copy genes and gtRNAdb annotations, for each sample, 3t-seq reports the number of reads covering each tRNA gene. Such counts are collated in a single matrix and differential expression is calculated using DESeq2 [8].

### Software dependencies management

In summary, 3t-seq integrates 18 different tools. The alternative to 3t-seq is the manual installation of each tool that is not only time-consuming but may also lead to version mismatches undermining analysis reproducibility. To address this issue, the pipeline leverages Snakemake’s integration with the Conda [44] package management system. Every step of the pipeline is associated with a specific Conda environment. Each Conda environment is declared in a file attached to the pipeline itself. In total, 12 Conda environment definitions are provided. SalmonTE, which is not available on Conda repositories, is provided as a Docker container.

This paradigm implies that the only mandatory dependencies of the pipeline are the Conda package management system and a Docker-compatible container engine (e.g. Singularity).

### Software execution

At first execution, the pipeline will create all necessary Conda environments, download SalmonTE Docker container, and convert it into a Singularity image. Subsequent runs will take advantage of the preconfigured environments and containers and run without re-installing them.

The pipeline supports Snakemake profiles, a mechanism to abstract computing environment-specific configurations. The pipeline is distributed with an example profile that specifies a list of common command line arguments and sets default resources for execution on an high performance computing (HPC) cluster environment, for example it sets the billing account and the default run time for single jobs, among the others. Nevertheless, each job has been tested, and its definition has been decorated with cluster resources allocation. Occasionally, jobs fail for unpredictable reasons, especially on distributed architectures. To handle this situation, fallible jobs have been implemented and coupled with dynamic resources allocation. Therefore, whenever a job fails, it gets restarted with an increased amount of resources and allows it to run longer.

## Results

### Output files

The pipeline outputs aggregate quality control reports from multiple stages of the processing: it produces a report for the input sequencing reads, their trimmed version, the subset of aligned reads, and the remaining ones after duplicates removal. All these reports are produced in HTML format and contain interactive plots, if <100 samples are given.

The mapping procedure for single-copy genes generates alignment files in genomic coordinates. These are converted into bigwig format to allow easy exploration with standard genome browsers. Mapping with TE-specific parameters (STARTE methods) generates transcriptome-aligned files. In all cases, splicing junction quantification is generated, and the pipeline outputs mapping statistics that are collected and reported to the user together with reports mentioned above and aligned reads statistics.

All gene expression quantification procedures (STAR for single-copy genes, the two STARTE methods, and tRNA method) generate plain text files containing read count matrices. For single-copy genes, STARTE-random, and tRNA, the pipeline runs a DESeq2 analysis to identify differentially expressed genes, and it delivers a binary representation of the DESeq2 object containing both raw and processed read counts. In addition, tables with ${\log}_2$ fold changes, ‘*P-*values’ and statistics are delivered. For STARTE-multihit, it was not possible to run DESeq2 because the estimated gene expression values are not represented with integer numbers. This violates the basic assumptions of the DESeq2. For single-copy genes, ${\log}_2$ fold change shrinkage was implemented. The resulting table is delivered to end users alongside the one carrying regular ${\log}_2$ fold change values. We refer to the DESeq2 manual for a detailed description of the shrinkage method and its use cases.

The SalmonTE module produces read count matrices aggregated at the clade and class level. It also produces a plain text table holding differential expression testing results and delivers an RData file containing R objects used during the analysis. In addition, it produces an MA plot to show the relationship between mean expression value and ${\log}_2$ fold change, as well as clade and class-level box plots comparing TE expression in the two tested conditions.

**Figure 2:**
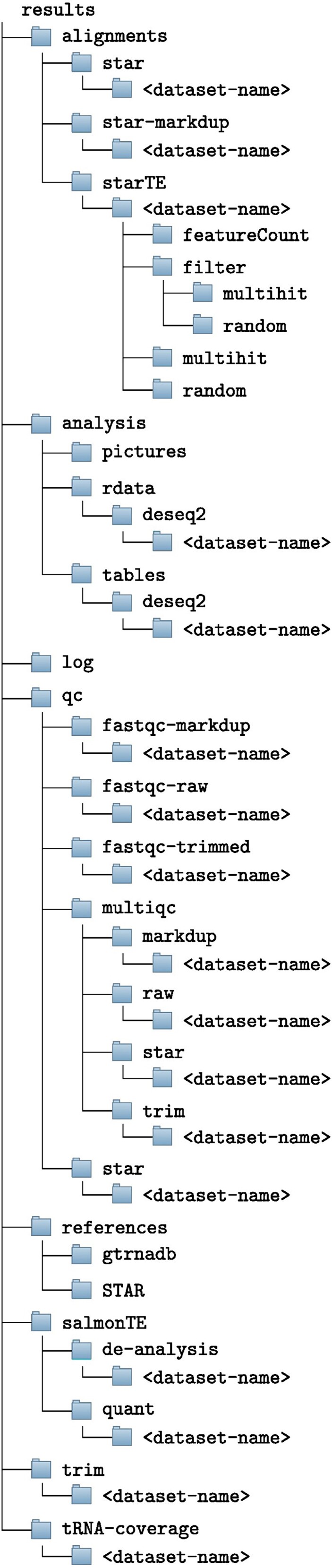
Example of the folder tree generated by 3t-seq. Results for each processed dataset are separated; here, the <dataset-name> placeholder represents the name of each input dataset. Only folders are reported.

The pipeline’s outputs are stored on the file system in a folder tree organized by experiment ID (Fig. 2). Results are split into several folders:

- qc: holds quality control reports from FastQC and MultiQC;
- trim: contains trimmed reads;
- alignments: contains all STAR outputs. Both standard and TEs-specific alignments can be found here. TEs quantification results can also be found here.
- analysis: contains figures and R objects in rds format for further analysis
- tRNA-coverage: contains the results of the tRNA coverage analysis
- salmonTE: contains all the results of SalmonTE including figures and R objects
- log: contains all log files organized by experiment name.

### Example use-case

This section demonstrates a use-case on a total RNA-seq dataset from E8.5 embryos with a demethylated genome caused by an inactivating mutation in *DNA methyltransferase 1* (*Dnmt1*, Gene Expression Omnibus (GEO): GSE130735) [45]. This dataset was used as a test case because *Dnmt1*-mutant embryos are known to release specific TEs from silencing [45, 46]. Data required to reproduce results hereafter described are included in the software package, with instructions on how to generate them.

### Preparing the samples sheet

Six mouse ribo-depleted total RNA-seq samples were downloaded from the GEO: three biological replicates for wild type and three for *Dnmt1* homozygous mutants. We created a sample sheet to summarize the relevant experimental variables and provide the pipeline with basic metadata (Table 1).

**Table 1:** Example sample sheet for 3t-seq

Sample	Filename_1	Filename_2	Genotype
SRX5795112_SRR9016958	SRX5795112_SRR9016958_1	SRX5795112_SRR9016958_2	WT
SRX5795113_SRR9016959	SRX5795113_SRR9016959_1	SRX5795113_SRR9016959_2	WT
SRX5795114_SRR9016960	SRX5795114_SRR9016960_1	SRX5795114_SRR9016960_2	WT
SRX5795117_SRR9016963	SRX5795117_SRR9016963_1	SRX5795117_SRR9016963_2	KO
SRX5795118_SRR9016964	SRX5795118_SRR9016964_1	SRX5795118_SRR9016964_2	KO
SRX5795119_SRR9016965	SRX5795119_SRR9016965_1	SRX5795119_SRR9016965_2	KO

In a nutshell, the sample column represents a human-readable name for each sample. Given the paired end sequencing protocol used to generate these data, two columns named filename_1 and filename_2 are included in the sample sheet. These represent file names holding the two mates stripped of file extensions (e.g. .fastq.gz). Full paths to these files will be computed at runtime from these values and values provided in the config file (see below).

If samples were sequenced with a single-end protocol, each sequencing library would have been composed of one single file. In this scenario, the sample sheet table can have a unique column called filename.

Finally, the genotype column reports the variable of interest. This column can have any name and should carry two levels. Multilevel contrasts are not supported at this stage.

To allow parallel processing of datasets generated with different sequencing protocols, each dataset can be described in a separate samples sheet and the pipeline configured to handle each of them independently.

#### Preparing the config file

Snakemake’s configuration files are written in YAML format; 3t-seq is no exception: 

**Table:** 

# config/config.yaml sequencing_libraries: -name: GSE13073 protocol: pe sample_sheet: sample-sheet.csv trimmomatic: >- "ILLUMINACLIP:TruSeq3-PE.fa:1:0:15:2 SLIDINGWINDOW:20:22 MAXINFO:20:0.6 LEADING:22 TRAILING:20 MINLEN:75" star: >- "--seedSearchStartLmax 30 --outFilterMismatchNoverReadLmax 0.04 --winAnchorMultimapNmax 40" bamCoverage: "--binSize 50 --normalizeUsing None" deseq2: test: Wald variable: genotype reference_level: WT globals: reads_folder:. results_folder: results/

**Table:** 

qc_folder: results/qc log_folder: results/log references_folder: results/references tmp_folder: /tmp analysis_folder: results/analysis genome: label: mm10 annotation_type: ensembl fasta_url: <Genome fasta URL> gtf_url: <Genome annotation URL> rmsk_url: <RepeatMasker annotation URL> gtrnadb_url: <GtRNADb bundle URL>

The configuration file is organized in three main sections:

1. sequencing_libraries: this section is a list of experiments to analyze. Each experiment must be associated with a name and sample sheet. Custom parameters for Trimmomatic, STAR, bamCoverage, and DESeq2 can be specified in the corresponding keys. Refer to each tool manual for all the necessary details.
2. globals: this section sets global variables, valid for all experiments. These parameters set paths to directories holding input, output, reference, log files, quality control outputs, and so on.
3. genome: this section defines genome parameters. All the experiments will be analyzed using the same genome; therefore, the pipeline expects all the experiments to be carried out in the same organism, at the very least. Given the path to the genome sequence file, the gene transfer format file for single-copy genes and TEs, and the GtRNAdb zip archive, the pipeline will download these files and store them locally in the path defined by the reference_folder key.

#### Running the pipeline

The pipeline can be run using Snakemake in a standard Unix command line. For example:

**Table:** 

$ snakemake --profile profile/default

The profile/default path points to a Snakemake profile folder. Such a folder contains a single config.yaml file that sets all the command line arguments necessary to run the pipeline. For example:

**Table:** 

# profile/default/config.yaml cores: 2 jobs: 1 reason: True printshellcmds: True software-deployment-method: -conda - singularity

These options configure Snakemake to use two cores; therefore, limiting processes requiring more cores (for example, STAR alignments) allow one single job to be executed at each time, effectively disabling Snakemake built-in parallelization, and force the pipeline to print the reason why it is executing a job and the shell command it is using to run it. This profile also forces the utilization of the Conda package manager and Singularity containers, enabling the creation of all the needed environments and downloading container images to run the pipeline. These last two options are required to run 3t-seq.

The command above assumes that Snakemake is available in the user environment. It is recommended to install the Snakemake tool using the Conda package manager in a new Conda environment. This can be achieved with a single command in a Unix shell where the conda command is available:

**Table:** 

$ conda create \ -n "snakemake-<version>"\ -c "conda-forge" \ -c "bioconda" \ "snakemake=<version>"

Upon creation, before utilization, any Conda environment must be activated:

**Table:** 

$ conda activate snakemake-<version>

With these elements in place, a computing environment is correctly configured to run the 3t-seq pipeline.

#### Collecting results

**Figure 3:**
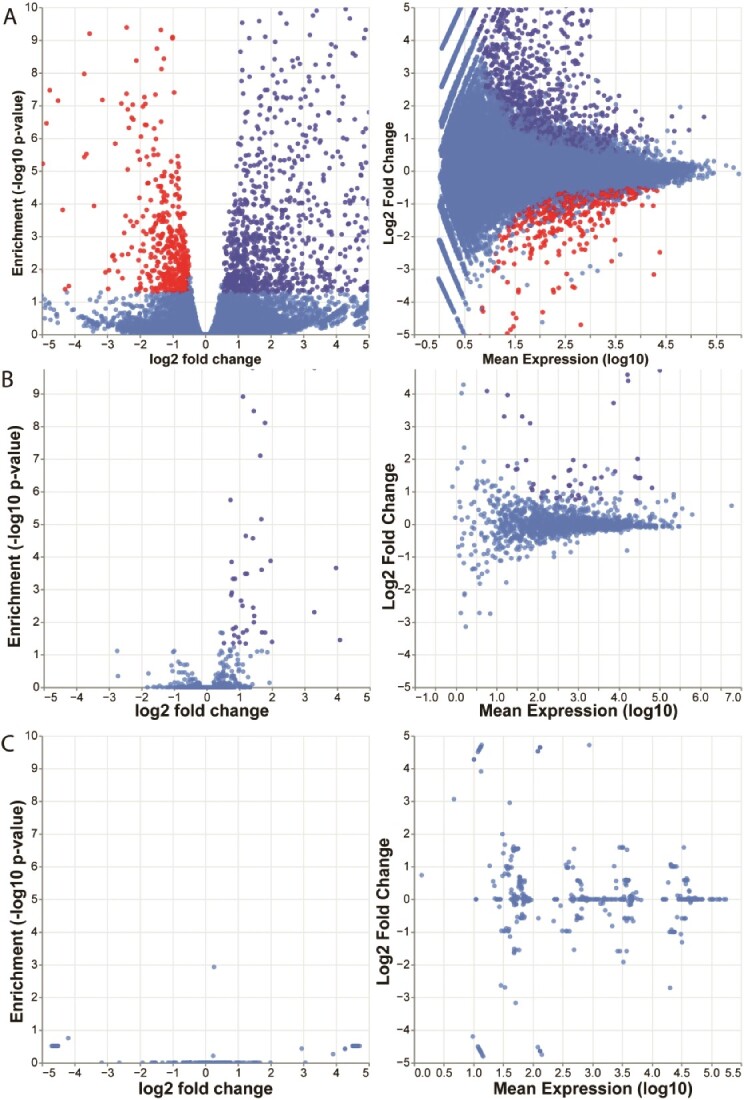
Visualizations of the results from differential expression analyses as reported in the interactive HTML report. The results of the test case comparing Dnmt1-null E8.5 embryos with wild-type littermate is shown as volcano plots and MA plots. (A) Single-copy gene. (B) TEs (starTE-random). (C) tRNA. As previously shown, Dnmt1-null embryos release specific TEs from silencing (B).

The pipeline is configured to generate a report with execution statistics and results. It collects executed steps, software versions, results from quality control, and differential expression analysis results. Figure 3 shows an example of the plots users can generate within the report. Plot parameters can be customized allowing fast results exploration. Tabular data used for visualization are provided in a separate view and can be readily saved to disk in a comma-separated format.

In addition to results, users can retrieve execution statistics on each step of the pipeline. These are presented as a directed acyclic graph showing how each step connects to others. By selecting any step, users can get information on software packages, input and output files, and the code executed within a particular pipeline step. Pipeline run statistics are presented visually in dotplots summarizing how long each step took and when it was run.

Results within the HTML report are organized in multiple sections. Within each section, results for each dataset are reported:

- FastQC section: collects FastQC reports for single samples collected at multiple steps of the pipeline, specifically: raw reads, trimmed reads, aligned reads (single-copy genes), and deduplicate alignments.
- MultiQC section: reports FastQC-aggregated reports at the same checkpoints of the pipeline execution.
- Single-copy genes section: reports differential expression statics as a table with and without shrinking ${\log}_2$ fold change (as per DESeq2). Two additional views report the volcano plot and MA plot generated from the same results.
- StarTE section: reports results for a differential expression analysis run on starTE-random read counts. The ${\log}_2$ fold change is computed only with the DESeq2 default method. A volcano plot and an MA plot are provided similarly to single-copy genes.
- tRNA expression section: reports similar results to the previous two sections. The ${\log}_2$ fold change is computed only with the default DESeq2 method, similarly to the starTE section.
- SalmonTE section: reports the results of differential expression analysis as implemented in the SalmonTE package. The package delivers three figures showing TE expression changes in one condition with respect to another.

The online repository contains an example dataset, configuration files, and pipeline invocation to generate an example report.

## Discussion and conclusions

The analysis of RNA-seq data has been standardized and implemented extensively over the last decade. However, we noted the absence of a comprehensive tool to study the expression of three important RNA species: single-copy genes, TEs, and tRNA. To our knowledge, there is no tool available to address this question in an integrated manner.

The 3t-seq pipeline implements standard, state-of-the-art data-handling procedures and provides a basis for a reproducible, portable, and scalable analysis. It implements a standard workflow of differential gene expression analysis, two reference genome-mapping-based TEs quantification methods, one alignment-free TEs method, and one tRNA-specific method. The pipeline is capable of downloading and re-using the same reference files across multiple runs, which allows for readily comparable analysis and minimizes differences across runs.

The Snakemake framework allows the management of dependencies conveniently abstracting complicated computational environment setup procedures, allowing users to focus on their biological questions.

The pipeline was tested on mouse genome; however, provided that genomic sequence and annotation files are available, there is no organism limitation.

**Key Points**

- 3t-seq is a Snakemake pipeline for RNA-seq analysis that performs differential expression of three types of RNA: transcripts from single-copy genes, TEs-derived transcripts, and tRNAs.
- 3t-seq implements three different methods to analyze TEs expression changes and uses a specialized resource for tRNA coverage analysis.
- 3t-seq results are delivered in the form of an interactive HTML report with quality control, tabular data, and interactive visualizations.
